# An interaction of heart disease-associated proteins POPDC1/2 with XIRP1 in transverse tubules and intercalated discs

**DOI:** 10.1186/s12860-020-00329-3

**Published:** 2020-12-01

**Authors:** Ian Holt, Heidi R. Fuller, Roland F. R. Schindler, Sally L. Shirran, Thomas Brand, Glenn E. Morris

**Affiliations:** 1 Wolfson Centre for Inherited Neuromuscular Disease, RJAH Orthopaedic Hospital, Oswestry, SY10 7AG UK; 2 School of Pharmacy and Bioengineering, Keele University, Keele, ST5 5BG UK; 3 Imperial Centre of Translational and Experimental Medicine, National Heart and Lung Institute, Imperial College, 4th Floor, Du Cane Road, London, W12 0NN UK; 4 BSRC Mass Spectrometry and Proteomics Facility, University of St Andrews, North Haugh, St Andrews, Fife, KY16 9ST UK

**Keywords:** Popeye domain-containing, Xin actin binding repeat-containing, Cardiac conduction, Intercalated discs, Transverse tubules

## Abstract

**Background:**

Popeye domain-containing proteins 1 and 2 (POPDC1 and POPDC2) are transmembrane proteins involved in cyclic AMP-mediated signalling processes and are required for normal cardiac pacemaking and conduction. In order to identify novel protein interaction partners, POPDC1 and 2 proteins were attached to beads and compared by proteomic analysis with control beads in the pull-down of proteins from cultured human skeletal myotubes.

**Results:**

There were highly-significant interactions of both POPDC1 and POPDC2 with XIRP1 (Xin actin binding repeat-containing protein 1), actin and, to a lesser degree, annexin A5. In adult human skeletal muscle, both XIRP1 and POPDC1/2 were present at the sarcolemma and in T-tubules. The interaction of POPDC1 with XIRP1 was confirmed in adult rat heart extracts. Using new monoclonal antibodies specific for POPDC1 and POPDC2, both proteins, together with XIRP1, were found mainly at intercalated discs but also at T-tubules in adult rat and human heart.

**Conclusions:**

Mutations in human *POPDC1*, *POPDC2* and in human *XIRP1*, all cause pathological cardiac arrhythmias, suggesting a possible role for POPDC1/2 and XIRP1 interaction in normal cardiac conduction.

**Supplementary Information:**

The online version contains supplementary material available at 10.1186/s12860-020-00329-3.

## Background

Popeye domain containing protein 1 (POPDC1), also known as blood vessel epicardial substance (BVES), was first found in chicken heart by subtractive hybridisation [1, 2]. *Popdc1* and two related gene family members, *Popdc2* and *Popdc3*, were identified in mammals and shown to be developmentally regulated and preferentially expressed in cardiac and skeletal muscle [2]. Human *POPDC1* is found on chromosome 6q21 along with *POPDC3* in tandem array, whereas *POPDC2* is found on human chromosome 3q13.33. The POPDC proteins are highly conserved throughout the animal kingdom, suggesting that they play an essential role [3].

POPDC proteins consist of a short extracellular N-terminal sequence which is glycosylated, three transmembrane domains, a conserved intracellular Popeye domain and a variable C-terminal domain which is isoform-specific, contains regions of low complexity and may be phosphorylated [4]. POPDC1 exists at the plasma membrane as a homodimer, which is stabilised by disulphide bonds [5, 6]. The predicted secondary structure of the Popeye domain contains a cyclic nucleotide binding domain, which binds the second messenger cyclic adenosine 3′,5′-monophosphate (cAMP) with high affinity [7]. Interaction between POPDC proteins and the potassium two pore domain channel subfamily K member 2 (KCNK2, also known as TREK-1) has been demonstrated, which leads to an increase in KCNK2 current in isolated mouse sinus node myocytes, and this activity was reduced by an increase in cAMP levels [7]. A number of other membrane proteins have been reported to interact with POPDC proteins, including caveolin-3 (CAV3) in mouse cardiomyocytes, which is a major component of caveolae in striated muscle membranes [8].

A homozygous missense variant in *POPDC1* has been found in a family with cardiac arrhythmia and limb-girdle muscular dystrophy (LGMD). This autosomal recessive mutation in *POPDC1* is associated with reduced cAMP affinity [9]. More recently, three homozygous loss-of-function mutations in *POPDC1* were identified in three families with LGMD and cardiac conduction abnormalities [10] and a missense mutation in *POPDC1* was observed in a patient with contractures and possible mild cardiac involvement [11]. A heterozygous nucleotide substitution in *POPDC2* has been associated with severe atrioventricular block [12] and homozygous missense variants in *POPDC3* have been associated with limb girdle muscular dystrophy in the absence of a cardiac phenotype [13]. POPDC1 protein was down-regulated with abnormal immunolocalisation in failing human hearts and POPDC1 and POPDC3 mRNA levels were reduced in the left ventricles of end-stage failing hearts [14]. *Popdc1* null mice showed impaired skeletal muscle regeneration [15] and increased sensitivity towards ischemia reperfusion [8]. Moreover, mice with null-mutations in *Popdc1* or *Popdc2* developed a stress-induced sinus node bradycardia due to pacemaker dysfunction [7, 16]. Knockdown of *popdc2* in zebrafish by injecting embryos with morpholino oligonucleotides resulted in the aberrant development of skeletal muscle and heart. A reduction in oligonucleotide concentration lead to an improvement in the skeletal muscle pathology, but abnormalities in the cardiac conduction system remained, resulting in cardiac arrhythmia and a reduction in heart rate [17].

Immunolocalization studies with polyclonal antibodies have shown that POPDC1 and POPDC2 generally localise to the sarcolemma of control skeletal muscle, but this membrane localisation was greatly reduced in muscle tissue from patients with pathogenic mutations in *POPDC1* [9, 10]. In the heart, POPDC1 and POPDC2 were found at the plasma membrane of cardiomyocytes, with high levels in the cardiac conduction system [7, 18].

In addition to the essential roles that POPDC proteins play in the maintenance of structure and function of skeletal muscles and in cardiac pacemaking and conduction, POPDC1 may play a role in tumor formation [19]. POPDC1 is thought to have a tumor suppressor function and decreased POPDC1 expression due to DNA methylation occurs in the early stages of a number of cancers [reviewed: [20, 21]].

Here we report a proteomic study to identify novel POPDC1/2-interacting proteins. The most significant hit with both POPDC1 and POPDC2 was XIRP1 (Xin actin binding repeat containing 1, also known as Xin or cardiomyopathy-associated protein 1 (CMYA1)). Pull-down and co-immunoprecipitation experiments confirmed the interaction of XIRP1 with POPDC1 and POPDC2. POPDC1/2 and XIRP1 displayed colocalization at the intercalated disk and in T-tubules. Mutations for both genes in model organisms [7, 17, 22, 23] and patients [9, 10, 24] have been associated with severe cardiac conduction defects and impaired skeletal muscle regeneration was reported for both *Popdc1* [15] and *Xirp1* [25, 26] null mutants. This suggests some overlapping function between POPDC1 and XIRP1, which could be based in part on the interaction reported here.

## Results

### XIRP1 and cardiac actin are the highest-scoring binding partners for both POPDC1 and POPDC2 in skeletal muscle myotubes

With the goal of identifying novel protein interaction partners for POPDC proteins in skeletal muscle, POPDC proteins available as GST-fusion “bait” proteins (mouse POPDC1 or POPDC2) were attached to glutathione beads and used to pull-down proteins from RIPA extracts of human skeletal myotubes. Proteins pulled down by the beads were identified by mass spectrometry (unfiltered results shown in: Additional file 1). Cardiac actin and XIRP1 had the highest scores for a number of significant peptides identified by mass spectrometry (Table 1) for both mouse POPDC1 and mouse POPDC2, after eliminating non-specific binding of proteins to control beads (no fusion protein attached). Annexin A5 was also present in both pull-downs. For further study, we selected from Table 1 those proteins with known involvement in cardiac conduction and / or cardiomyopathy: XIRP1 and annexin A5.

**Table 1:** Mass spectrometry abridged results of mouse POPDC1/2 pull-downs

	Accession number	Number of significant (unique) peptides	
		mPOPDC1	mPOPDC2
Actin, alpha cardiac muscle 1	ACTC_HUMAN	12	12
Xin actin-binding repeat-containing protein 1	XIRP1_HUMAN	11	19
Tubulin beta-4B chain	TBB4B_HUMAN	9	7
Tubulin beta chain	TBB5_HUMAN	9	6
Tubulin beta-6 chain	TBB6_HUMAN	9	4
ATP synthase subunit beta, mitochondrial	ATPB_HUMAN	7	5
Fructose-bisphosphate aldolase A	ALDOA_HUMAN	5	7
L-lactate dehydrogenase A chain	LDHA_HUMAN	5	5
Histone H2AX	H2AX_HUMAN	4	4
PDZ and LIM domain protein 3	PDLI3_HUMAN	3	5
Glucosidase 2 subunit beta	GLU2B_HUMAN	3	5
Lamina-associated polypeptide 2 (beta/gamma)	LAP2B_HUMAN	3	4
Histone H2A.V	H2AV_HUMAN	3	4
Phosphoglycerate mutase 1	PGAM1_HUMAN	3	4
Phosphoglycerate kinase 1	PGK1_HUMAN	3	4
Heat shock protein beta-2	HSPB2_HUMAN	3	4
Annexin A5	ANXA5_HUMAN	3	3
40S ribosomal protein S30	RS30_HUMAN	3	3

Only proteins that met the following criteria are shown: identified from more than two significant unique peptide sequences, undetected in the control pull-down AND detected across both mPOPDC1 and mPOPDC2 pull-downs. See Additional file 1 for full results

Cardiac actin is the principal actin isoform during early stages of skeletal myogenesis and is only gradually replaced by skeletal muscle actin in adult skeletal muscle [27], so it is not surprising that the cardiac isoform was detected in myotube extracts.

### Confirmation by western blotting of POPDC1 interaction with XIRP1 in extracts of both human myotube cultures and adult rat heart

**Fig. 1:**
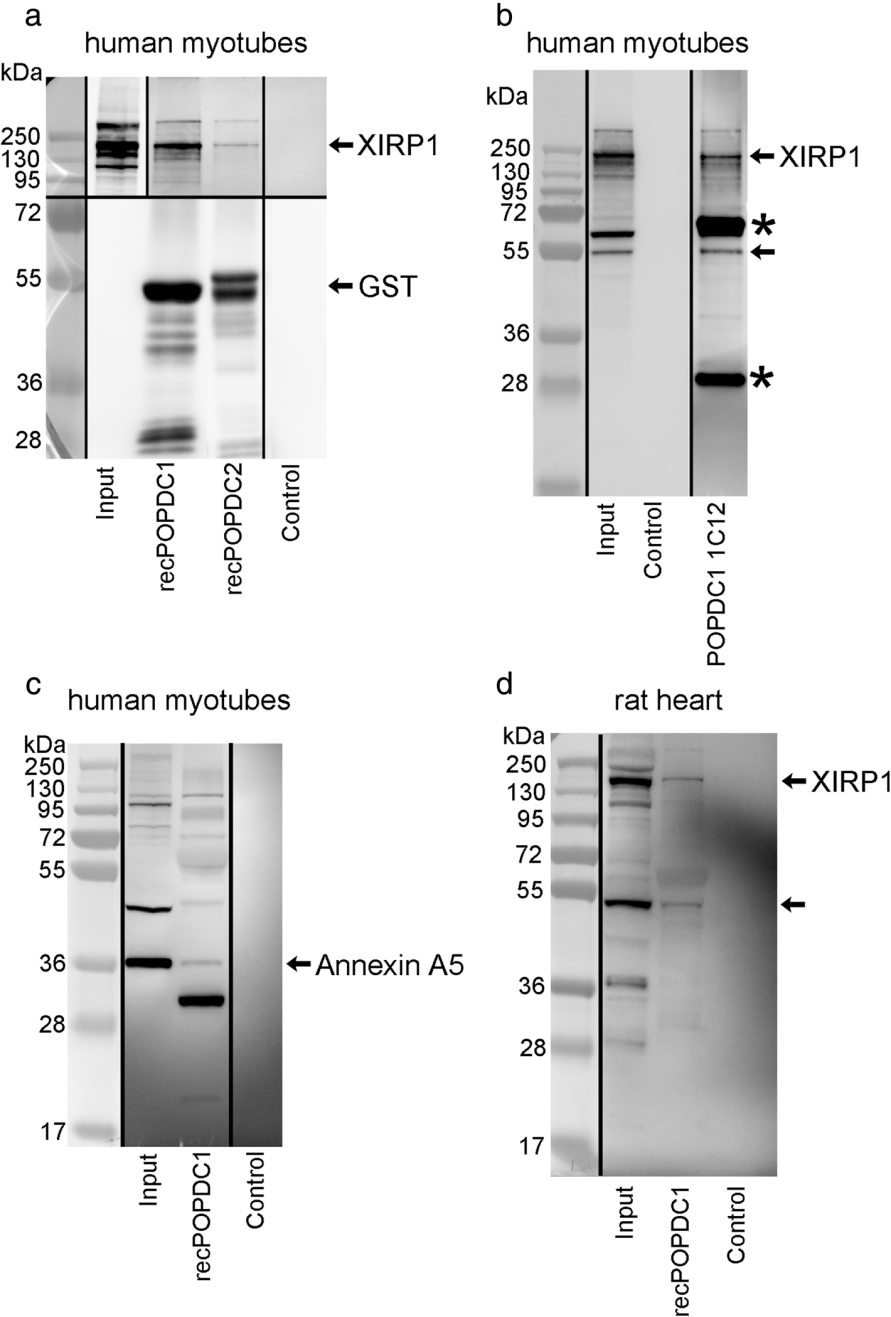
POPDC proteins pull down and co-immunoprecipitate XIRP1 and pull down annexin A5 from human myotube extract and pull-down XIRP1 from rat heart extract. Vertical black lines separate parts of blots that were originally non-adjacent or with different exposure times, as described. In all cases, the molecular weight markers in lane 1 were detected by colourimetric imaging and the antibody blots by chemiluminescence. **a** Glutathione beads were loaded with recombinant preparations of POPDC1, POPDC2 or not loaded (control for non-specific binding) and incubated with human myotube extracts. “Input” shows human myotube extracts diluted to 20%. Upper part of blot (A) (above 72 kDa) developed with mAb against XIRP1 (sc-166,658) shows XIRP1 in the input and pulled down by POPDC1 and POPDC2, but not in the control. The “Input” lane had a shorter exposure time than the others and the “Control” lane was originally non-adjacent. The Lower part of blot (A) (72 kDa and below) was from a separate but identical gel to the upper blot and was developed with mAb against GST tag (17A10) as a loading control. The “Control” lane was originally non-adjacent to the others. The predicted molecular weights of the Popdc1 and Popdc2 fusion proteins are 58 and 57 kDa respectively, which are indicated approximately with a single arrow. **b** Pan mouse IgG Dynabeads were loaded with monoclonal antibody against POPDC1 (1C12) or not loaded (control) and incubated with human myotube extracts. The blot was developed with XIRP1 mAb and shows XIRP1 in the input and immunoprecipitated by the POPDC1 mAb but not by unloaded control beads. * = mouse Ig H and L chains. Predicted molecular weights of human XIRP1, isoforms 1,2 and 3 are 199, 122 and 56 kDa respectively. Arrows indicate bands corresponding to XIRP1 isoforms 1 and 3. The “POPDC1 1C12” lane had a longer exposure compared to the other lanes and the “molecular weight markers” were not originally in lane 1. **c** Glutathione beads loaded with recombinant POPDC1 or beads alone (control) were incubated with human myotube extract. The blot was developed with pAb against annexin A5. The arrow indicates a band corresponding to annexin A5 monomer (36 kDa) in the input and POPDC1 pull-down, but absent from the non-specific binding control. The “Control” lane was originally non-adjacent to the others. **d** Glutathione beads loaded with recombinant POPDC1 or beads alone (control) were incubated with rat heart ventricle extract. The blot was developed with mAb against XIRP1. Arrows indicate bands corresponding to XIRP1 in the input and POPDC1 pull-down, but absent from the non-specific binding control. The original colourimetric and chemiluminescence images are shown in Additional File 2

The presence of XIRP1 in POPDC1/2 pull downs was confirmed by western blotting with a commercial anti- XIRP1 antibody (Fig. 1a upper blot). Figure 1a lower blot is a loading control showing antibody against the GST “bait” protein. XIRP1 was also immunoprecipitated from the same human myotube extracts by mAb 1C12 against POPDC1 (Fig. 1b). Western blots are representative of pull-downs repeated using three separate extracts of the cultured myotube cell line. Recombinant POPDC1 attached to glutathione beads also pulled-down XIRP1 from a RIPA extract of adult rat heart ventricle (Fig. 1d). The pull down of annexin A5 by recombinant POPDC1 from myotubes was also confirmed by western blotting (Fig. 1c). (Uncropped blots shown in: Additional file 2).

### New monoclonal antibodies against POPDC1 and POPDC2

**Fig. 2:**
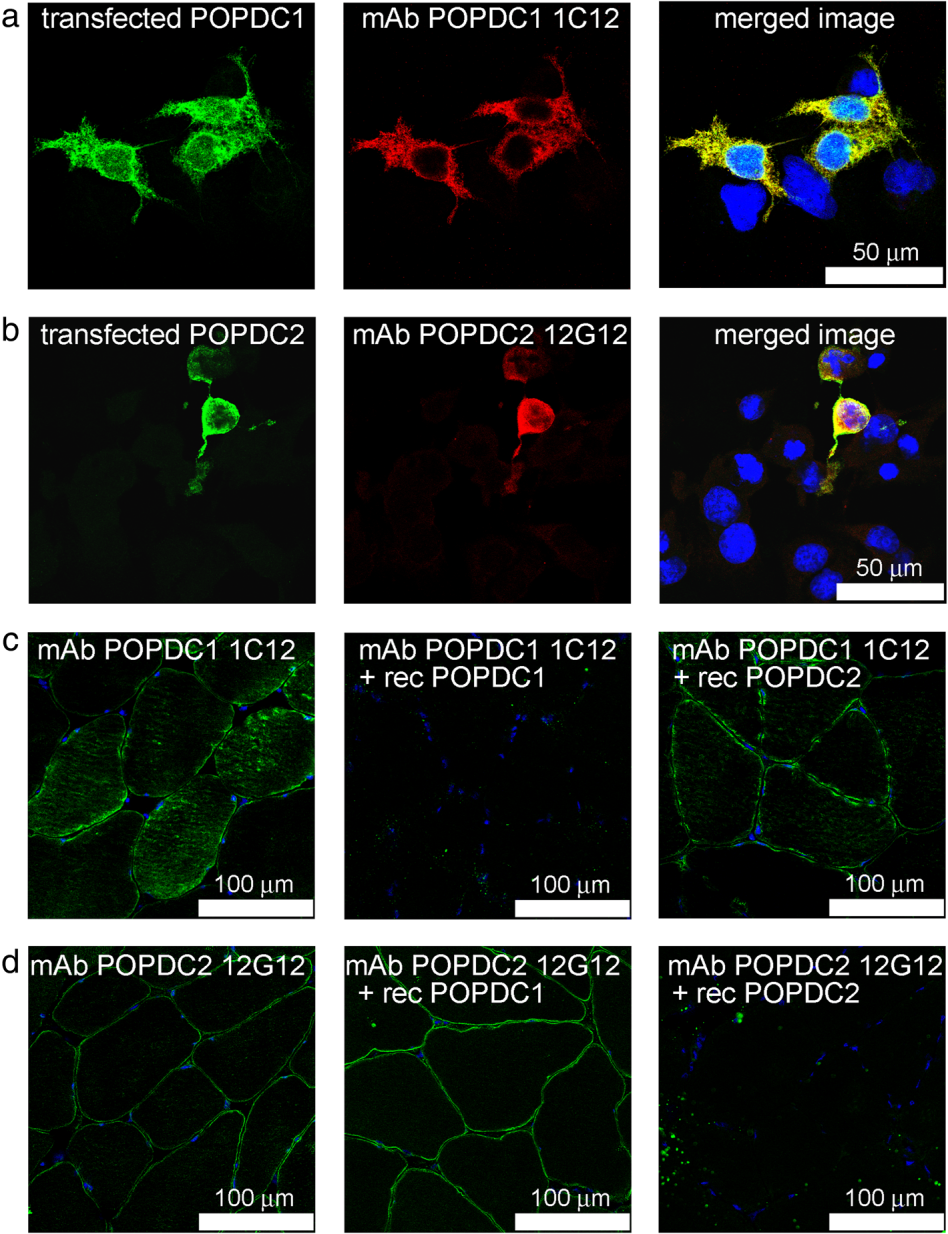
Immunofluorescent specificity of POPDC mAbs. COS-7 cells were transfected with either (**a**) human POPDC1 (GFP tag) or (**b**) human POPDC2 (FLAG tag) and processed as described in “methods”. **a** mAb POPDC1 1C12 colocalised with transfected POPDC1 and (**b**) mAb POPDC2 12G12 colocalised with transfected POPDC2. The POPDC1 mAb did not recognise transfected POPDC2 and the POPDC2 mAb did not recognise transfected POPDC1 (not shown). **c** mAb POPDC1 1C12 localised to the sarcolemma of human skeletal muscle sections. This localisation was prevented by pre-incubation of the mAb with recombinant POPDC1 but not when the mAb was pre-incubated with recombinant POPDC2. Also, **d** localisation of mAb POPDC2 12G12 to the sarcolemma was not inhibited by pre-incubation with recombinant POPDC1 but this localisation was inhibited when the mAb was pre-incubated with recombinant POPDC2

Monoclonal antibodies were prepared against recombinant protein preparations and selected for their recognition of human POPDC1 or POPDC2 proteins using techniques described previously [28]. Immunofluorescence specificity of the new POPDC1 1C12 (Fig. 2a) and POPDC2 12G12 (Fig. 2b) mAbs was shown by the staining of COS7 cells transfected with recombinant POPDC1 or POPDC2 respectively. POPDC1 1C12 did not recognise transfected POPDC2 and mAb POPDC2 12G12 did not recognise transfected POPDC1. Specificity of the mAbs was further demonstrated by competition experiments. Pre-incubation of mAb POPDC1 1C12 with recombinant POPDC1, but not with recombinant POPDC2, inhibited localisation of the mAb to the sarcolemma of human skeletal muscle (Fig. 2c). Similarly, pre-incubation of mAb POPDC2 12G12 with recombinant POPDC2, but not with recombinant POPDC1, inhibited sarcolemmal localisation (Fig. 2d).

### XIRP1 and POPDC1/2 are found, together with annexin A5, at intercalated discs and transverse tubules in rat or human heart sections

**Fig. 3:**
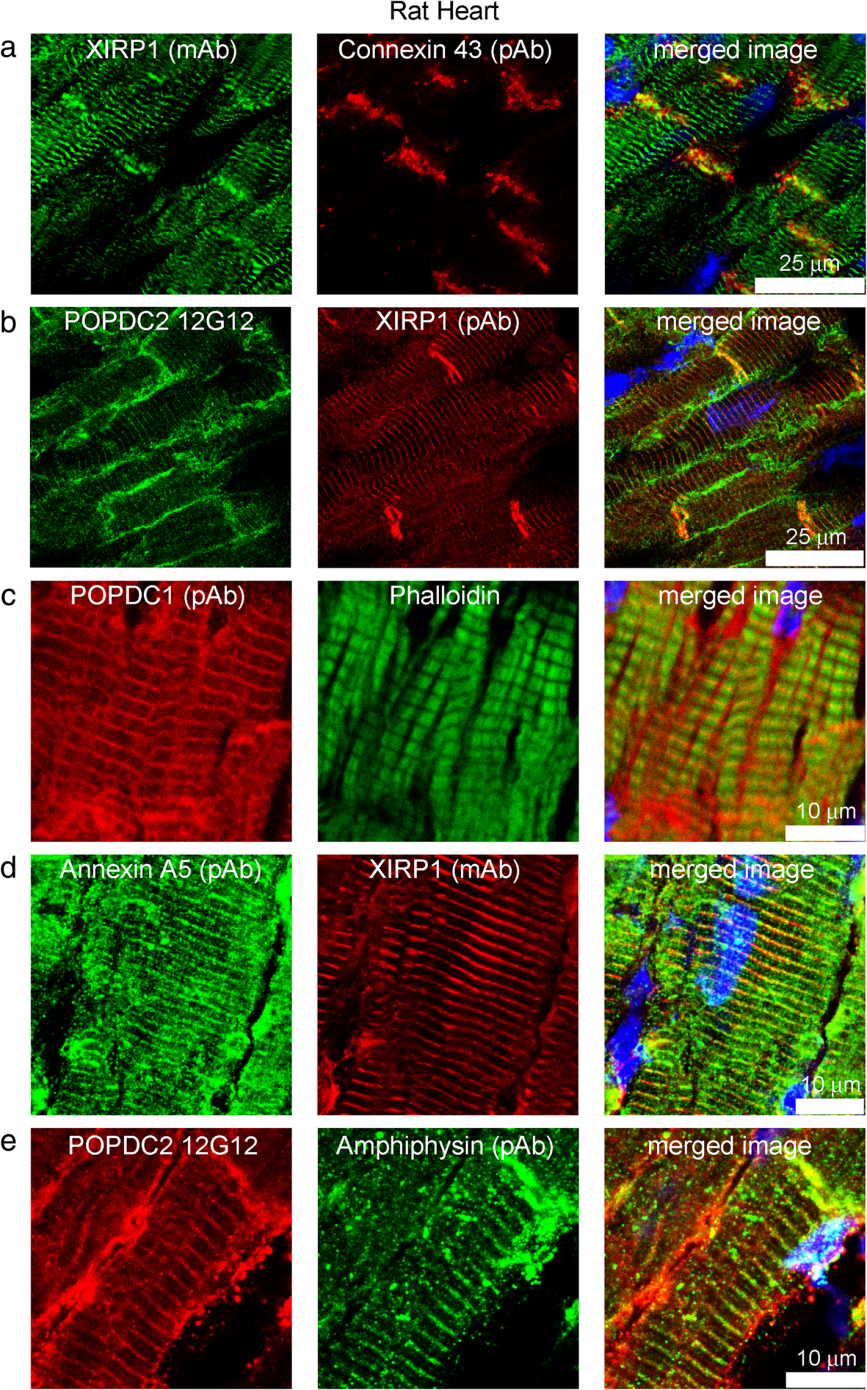
Immunolocalisation of POPDC1/2, XIRP1 and annexin A5 at intercalated discs and myofibrils in rat heart. In rat heart, intercalated discs were labelled with polyclonal antibody against connexin 43. **a** XIRP1 colocalised with connexin 43 at the intercalated disc and also stained myofibrils. **b** POPDC2 colocalises with XIRP1 at intercalated discs and myofibrils and POPDC2 is also present at the cardiac sarcolemma. **c** POPDC1 is similar to POPDC2, locating to intercalated discs and sarcolemma and to the centre of the actin I-band stained by phalloidin. **d** Annexin A5 colocalises with XIRP1. **e** POPDC2 colocalises with amphiphysin II, a marker for T-tubules

In rat heart, XIRP1 and POPDC2 were both present at intercalated discs and near the Z-line of myofibrils (Fig. 3a,b). Unlike XIRP1, however, POPDC2 was also present at the cardiac sarcolemma (Fig. 3b). A similar staining pattern in heart was reported by Alcalay et al. [8] for POPDC1 and Soni et al. [29] for POPDC2. Intercalated discs were identified using a connexin 43 antibody, which stains neither sarcolemma nor the Z-line (Fig. 3a). Our human-specific POPDC1 mAb does not recognize the rat protein, but a commercial POPDC1 antibody (BVES pAb STJ22848) gave similar results on rat heart to our POPDC2 mAb (Fig. 3c). Figure 3c also shows the location of POPDC1 near the Z-line which lies at the centre of the actin I-band stained by phalloidin (ALEXA 488 - phalloidin). This staining appears to correspond to transverse tubules, since an antibody against amphiphysin II, a marker for T-tubules, gave a similar staining pattern as POPDC2 (Fig. 3e) and POPDC proteins have transmembrane domains. Annexin A5 (ANXA5) was also present in both POPDC1 and POPDC2 pull downs (Table 1) and antibodies against this protein also located it close to the Z-line (Fig. 3d). Annexin A5 is a known component of the sarcolemma, transverse tubules and intercalated discs in the heart [30].

**Fig. 4:**
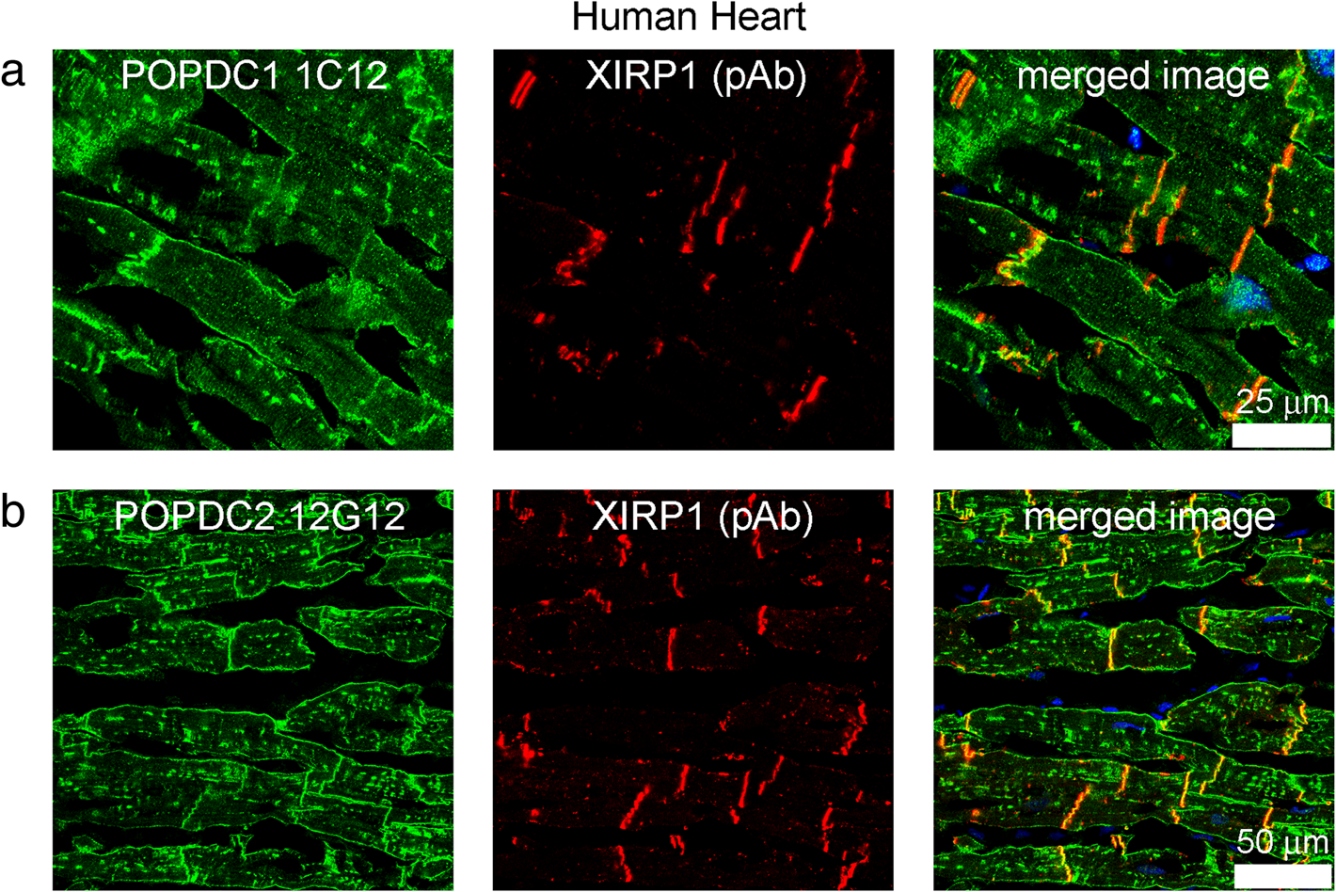
Immunolocalisation of POPDC1, POPDC2 and XIRP1 in human heart. **a** POPDC1 and **b** POPDC2 were present at the sarcolemma and at intercalated discs. XIRP1 was also present at intercalated discs. Cross striations were difficult to resolve in the human heart sections

In human heart, mAbs POPDC1 1C12 (Fig. 4a) and POPDC2 12G12 (Fig. 4b) stained the intercalated discs and the cardiac sarcolemma whereas XIRP1 was present at the intercalated discs only. There were similar staining patterns in rat and human hearts, but cross striations were difficult to resolve in the human heart sections. In low power microscopy (40x) of human heart, T-tubules were only faintly-stained (Fig. 4) compared with the higher power microscopy (63x) of rat heart (Fig. 3), suggesting that intercalated discs may be the major site of POPDC-XIRP1 interaction in the heart. Figures 3 and 4 show representative images of results that were obtained with sections from two different rat hearts and confirmed with the sections from a human heart.

### POPDC1, POPDC2, XIRP1 and annexin A5 are also found in T-tubules in longitudinal sections of human skeletal muscle

**Fig. 5:**
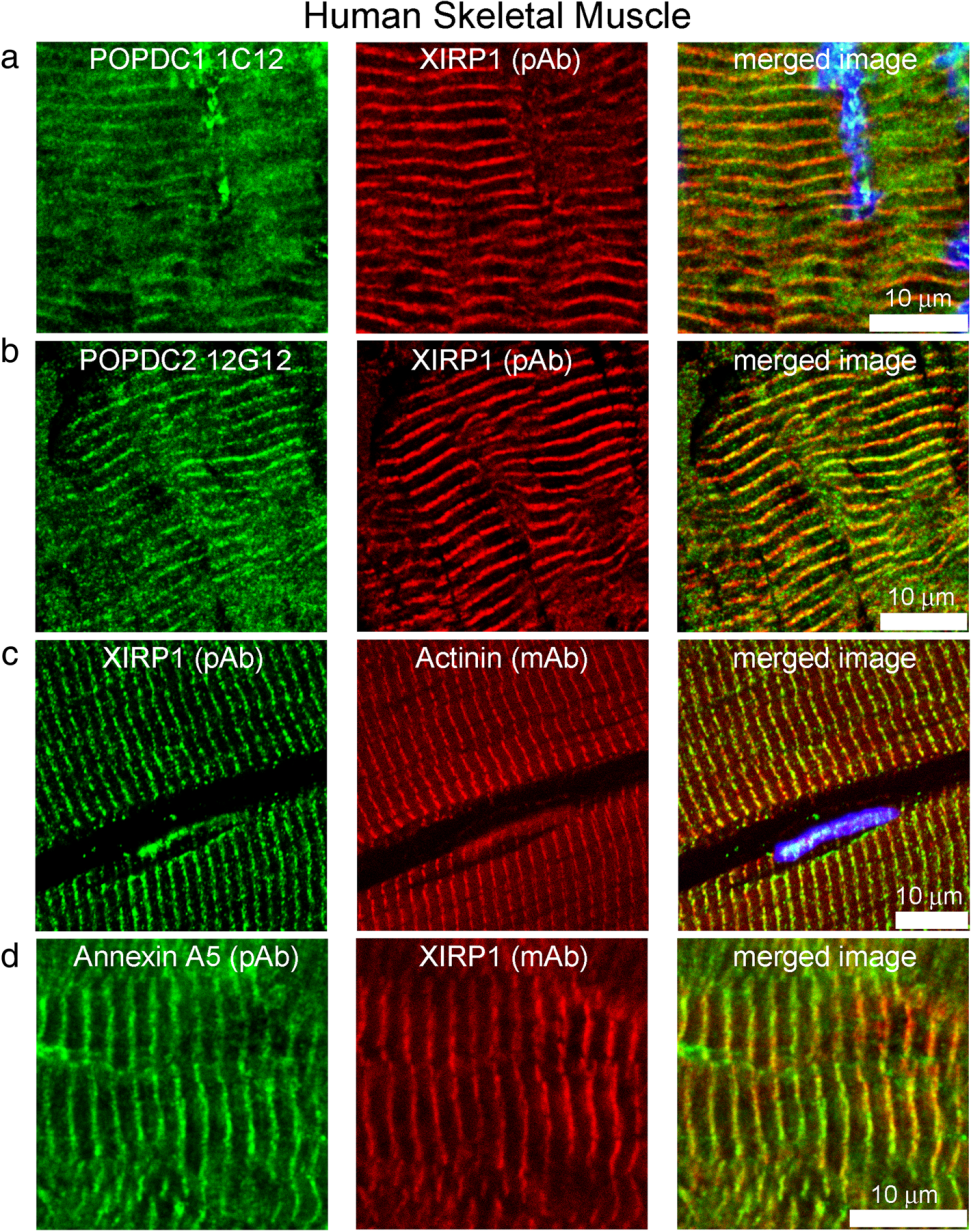
Immunolocalisation of POPDC1/2, XIRP1 and annexin A5 at myofibrils in longitudinal sections of human skeletal muscle. **a** POPDC1 and XIRP1, **b** POPDC2 and XIRP1, **c** XIRP1 and Actinin, **d** Annexin A5 and XIRP1, all colocalised at myofibrils

Figure 5a-d shows the co-localization of POPDC1 and 2, XIRP1 and annexin A5 at Z-lines/T-tubules in longitudinal sections of human skeletal muscle, using a monoclonal antibody against alpha-actinin to locate Z-lines (Fig. 5c).

### POPDC1, POPDC2 and XIRP1 are present at the sarcolemma in transverse sections of human skeletal muscle

**Fig. 6:**
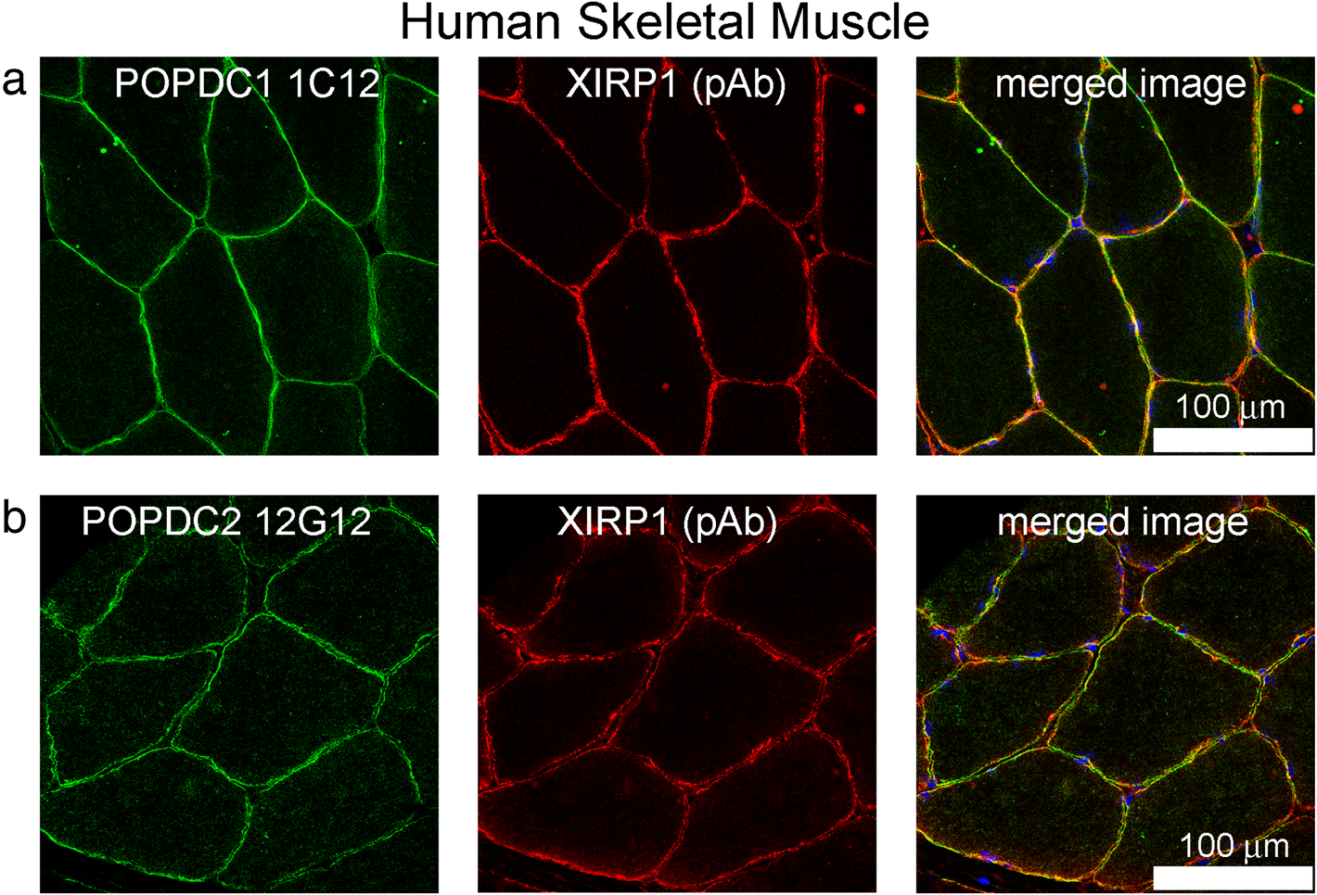
Immunolocalisation of POPDC1/2 and XIRP1 in transverse section of human skeletal muscle. **a** POPDC1 mAb 1C12 and **b** POPDC2 mAb 12G12 showed largely even and regular sarcolemmal staining whereas XIRP1 was often discontinuous at the sarcolemma

POPDC1 (Fig. 6a) and POPDC2 (Fig. 6b) are present at the sarcolemma in transverse sections of skeletal muscle. XIRP1 is also present at the sarcolemma, but does not co-localise exactly with POPDC proteins. Figures 5 and 6 show representative images of sections from five different human muscle biopsies which gave similar results in these experiments.

## Discussion

Our initial proteomic study of pull-downs from human myotube extracts using recombinant POPDC1 or POPDC2 as the bait proteins showed that XIRP1 and cardiac actin were the highest-scoring hits (Table 1). XIRP1 has been located to myotendinous junctions in skeletal muscle [22] and to intercalated discs in heart [31, 32]. We examined the interacting POPDC and XIRP1 proteins further by co-localization studies, bearing in mind that POPDC proteins (via zonula occludens-1, ZO-1 [33]) and XIRP1 [34] are known actin-binding proteins. Proteomic studies only give clues to potential interaction partners in a single tissue; Table 1 should not be expected to include all interaction partners of POPDC proteins and all potential partners revealed by proteomics require biochemical confirmation.

XIRP1 is a scaffolding protein which stabilizes and maintains the integrity of the intercalated disc. Overexpression of XIRP1 leads to increased phosphorylation of gap junction protein alpha 1 (GJA1), also known as connexin-43 (Cx43), and down-regulation of the activity of gap junctions in cardiomyocytes [35]). Knockout *Xirp1* (or Xinα) mice develop a late-onset cardiomyopathy with conduction defects [22]. Missense mutations in *XIRP1* cause cardiac arrhythmias [24] and a number of mutations in XIRP1 that are classed as deleterious have been found in patients with conditions including myalgia, proximal weakness, arrhythmia, contractures and cardiac conduction defects [11]. XIRP proteins have binding domains for both β-catenin and p120-catenin, through which they attach to N-cadherin in the intercalated disc [36]. Intercalated discs provide electromechanical coupling between adjacent cardiomyocytes. Depolarising current is transmitted via gap junctions in the intercalated discs from cell to cell across the heart. Defects in intercalated disc structure and function are common features of cardiomyopathy, arrhythmia and heart failure (Reviewed: [32]).

Annexin A5 was also a significant interaction partner for POPDC1/2 (Table 1). Annexin A5 is upregulated in failing human hearts [37] and has been linked to plasma membrane repair in skeletal muscle [38]), a property shared with caveolin-3 [39] and XIRP1 [40, 41]. There is some evidence of a role for POPDC1 in the maintenance of plasma membrane integrity, as discontinuities in the plasma membrane have been observed in the grandfather of a family carrying a pathogenic homozygous missense mutation (c.602C > T, p.S201F) in POPDC1 [9]. This mutation gives rise to cardiac arrhythmia and LGMD [9].

Using new monoclonal antibodies (mAbs) specific for either POPDC1 (1C12) or POPDC2 (12G12), together with a commercial polyclonal antibody against POPDC1 (BVES), we have shown that both proteins are localized at the sarcolemma and transverse tubules in both human skeletal muscle and rat heart muscle. Partial co-localization of both XIRP1 and annexin A5 with POPDC1/2 was found in T-tubules in both cardiac and skeletal muscles. T-tubules are invaginations in the plasma membrane of muscle cells, they are rich in ion channels and relay excitation signals from the external membrane to the interior of cells and are very sensitive to eccentric stretch (reviewed in [42]). In heart, POPDC1 and 2 are mainly concentrated at intercalated discs. The presence of POPDC2 at intercalated discs was also shown by Soni et al. 2016 [29], while co-localization of XIRP1 with Connexin-43 at the intercalated discs of human hearts was shown by Xie et al. 2017 [35]. There is considerable functional overlap between POPDC proteins and XIRP proteins. In null mutants in the mouse for both gene families, a cardiac arrhythmia phenotype was reported. In the case of *Popdc1* and *Popdc2* null mutants, a stress-induced bradycardia phenotype was observed, while at baseline the mutants showed a normal rhythmic beating heart [7]. In contrast, the *Xirp1* null mutant phenotype was characterized by a late-onset cardiomyopathy with conduction defects [22]. In patients, *POPDC1* has also been linked to prolonged QT interval, increased QRS duration and AV-block [43]. Interestingly, in contrast to the mouse, in zebrafish, an AV-block was seen for both *popdc1* and *popdc2* null mutants [9, 17, 43]. Functional overlap is also seen in *Popdc1* null mutants and *Xirp1* null mutants with regard to skeletal muscle regeneration [25, 26].

Although the pull-downs clearly show association between POPDC1/2, XIRP1 and actin, it is not clear whether the interactions between them are direct or indirect. It is possible that they are members of a larger complex, some components of which were not detected by proteomics for technical reasons. Earlier studies identified different binding partners for POPDC proteins, including dystrophin, dysferlin, VAMP2, VAMP3, GEFT, NDRG4, ZO-1, TREK-1; c-MYC and PR61-alpha, as well as caveolin-3 [7–9, 33, 44–47]. None of these are among the 340 proteins that appeared in our pull-downs, although this may be either because their interaction with POPDC is tissue-specific and not occurring in myotube cultures, or because they are present at very low levels in RIPA buffer extracts, or because they showed some non-specific binding to our control beads or because the interaction does not withstand the extraction conditions used. With numerous other interaction partners having been linked to POPDC1 and POPDC2 [4], it is not surprising that the loss-of-function phenotype of *POPDC1* and *XIRP1* would show only partial overlap. In this context it will also be interesting to define all components of the protein complex of which XIRP1 and POPDC1 are part in cardiac and skeletal muscle.

## Conclusions

POPDC1/2 proteins interact with XIRP1 in skeletal myotubes and adult heart. Determining the functions of POPDC and XIRP1 proteins at the t-tubules and intercalated discs will help to understand their roles in normal cardiac and skeletal muscle function and what can go wrong when pathogenic mutations occur.

## Methods

### Popdc “bait” proteins

GST fusion proteins were generated by PCR from cDNA for mouse *Popdc1* (*Bves,* GenBank Accession No: NM_024285.3) (aa 114 to 357) and for mouse *Popdc2* (GenBank Accession No: NM_001081984.2) (aa 98 to 339) and cloned into pGEX bacterial expression vectors. pGEX constructs were transformed into *E. coli* BL21(DE3) and induced by IPTG to give GST-fusion proteins. After incubation the cells were washed with TNE buffer and sonicated sequentially with TNE, 2 M urea, 4 M urea, 6 M urea and 8 M urea. A Coomassie Blue-stained gel showed that most of the recombinant proteins were extracted in the 6 M urea fraction and these were used in subsequent work. Resultant proteins contained GST-tags followed by the intracellular C-terminal tails of mPopdc1 and mPopdc2, with molecular weights of 58.3 and 57.1 kDa respectively.

### Muscle cell culture and extraction

A clonal immortalized human myoblast cell line from a 25-year-old control donor without neuromuscular disease was immortalized by transduction with human telomerase reverse transcriptase (hTERT) and cyclin-dependent kinase-4 (Cdk4) containing retroviral vectors, at the Institut de Myologie, Paris, as described previously [48]. Myoblasts were cultured in skeletal muscle cell growth medium (Cat No: C-23060; PromoCell GmbH, Heidelberg, Germany) containing supplement mix (Cat No: C-39365; PromoCell) with 20% Fetal Bovine Serum (Cat No: 10270; Gibco; ThermoFisher Scientific, Paisley, UK). At around 80% confluency, adherent myoblasts were washed in medium lacking serum and differentiation was induced by culturing in DMEM (Cat No: 31966–021; Gibco; ThermoFisher Scientific) supplemented with Insulin (1721 nM), Transferrin (68.7 nM), Selenium (38.7 nM) (ITS-X; Cat No: 51500–056; Gibco; ThermoFisher Scientific) and Penicillin-Streptomycin (Cat No: DE17-603E; Lonza; Verviers, Belgium). Cell culture was continued for a further 4 days, after which, over 80% of the cells had fused into myotubes. Cells cultured for pull-down experiments were extracted in RIPA buffer (1% NP-40; 0.25% sodium deoxycholate; 1 mM EDTA; 150 mM NaCl; 50 mM Tris-HCl, pH 7.4), in the presence of protease inhibitor cocktail (Sigma P8340; plus 1 mM PMSF), by sonication followed by centrifugation.

### Pull-down and proteomic analysis

Glutathione high capacity magnetic agarose beads (Cat No: G0924; Sigma) were loaded with GST-tagged “Bait” proteins (mouse Popdc1 or mouse Popdc2 or No Bait control) and then incubated with “Prey” (cultured human myotube extract in RIPA buffer). Captured proteins were digested by overnight incubation with 1% v/v trypsin at 37 °C and analysed by nanoLC MSMS as described previously [49]. The MS/MS data file was analysed using the Mascot search algorithm (Matrix Science), against the SwissProt database (May 2018) restricting the search to *Homo sapiens* (557,491 sequences), with trypsin as the cleavage enzyme and methionine oxidation as a variable modification. The peptide mass tolerance was set to 20 ppm and the MSMS mass tolerance to ±0.1 Da. This analysis was used to identify medium-to-high abundance proteins that interact with Popdc proteins.

### Extraction and pull-down with rat heart

The heart was removed from a 5 month old Sprague Dawley rat that had been euthanized humanely with an overdose of pentobarbitone anesthetic (0.5 mL/100 g) via intraperitoneal injection. The heart was cut in half, washed with PBS, weighed, snap frozen in liquid nitrogen, transferred to a mortar and ground to a fine powder with a pestle. The liquid nitrogen was then allowed to evaporate and the powdered heart extracted with RIPA buffer with inhibitors (around 1 mL buffer per 100 μg heart), sonicated on ice and centrifuged. The heart extract was used instead of myotube extract as “prey”, with mouse GST- mouse Popdc1 on glutathione beads as “bait”, as described earlier.

### Hybridoma production

Human *BVES* (*POPDC1*) cDNA (GenBank Accession No: NM_007073.4) was a gift from D. Bader [33]. A GST fusion protein was generated by PCR from the C-terminal tail of (aa 115–347) and cloning into the pGEX bacterial expression vector. This protein contains the GST tag followed by the intracellular C-terminal tail of POPDC1. The GST-mouse Popdc2 (amino acids 98 to 339) fusion protein preparation described earlier for pull-down experiments was also used for hybridoma production. Induction and extraction of the GST-fusion proteins was performed as described earlier. Fusion protein in the 6 M urea fraction was used in the protocol for immunization of BALB/c mice for production of mAbs [28]. ELISA plates were coated with either the GST-POPDC fusion proteins used for immunization or with an unrelated GST-fusion protein, in order to eliminate those mAbs reacting with the GST fusion tag. Hybridoma supernatants were additionally screened for colocalisation with transfected human POPDC proteins as follows: COS-7 cells were transfected (Lipofectamine) with either hPOPDC1-YFP or hPOPDC2-FLAG tag on glass coverslips and the cells fixed and permeabilized with 50% acetone: 50% methanol. Transfected cells were incubated with hybridoma supernatants. hPOPDC1-YFP transfectants were then incubated with 5 μg/ml goat anti-mouse ALEXA 546 (Cat No: A11030, Molecular Probes, Eugene, Oregon, USA). hPOPDC2-FLAG transfectants were incubated with rabbit oligoclonal anti-FLAG (Cat No: 740001, Thermo Fisher Scientific), followed by 5 μg/ml goat anti-mouse ALEXA 546 (Cat No: A11030, Molecular Probes) and 5 μg/ml goat anti-rabbit ALEXA 488 (Cat No: A11034, Molecular Probes). DAPI was added for the final 10 min of incubation to counterstain nuclei and confocal microscopy performed as described below. Monoclonal antibodies POPDC1-1C12 and POPDC2-12G12 were selected because they recognized the corresponding transfected POPDC1 or POPDC2 by immunofluorescence microscopy (Fig. 2).

### SDS-polyacrylamide gel electrophoresis and Western blotting

Samples were mixed with SDS buffer (125 mM Tris pH 6.8; 2% SDS; 5% 2-beta mercaptoethanol; 5% glycerol; with bromophenol blue), boiled and subjected to SDS-PAGE using 10% polyacrylamide gels and transferred to nitrocellulose membranes (Protan BA85, Whatman). Non-specific sites were blocked with 5% skimmed milk protein, membranes washed with PBS and then incubated with primary monoclonal antibodies against: XIRP1, (Xin-alpha (D-8); sc-166,658; 1/100; Santa Cruz Biotechnology, Insight Biotechnology Ltd., Wembly, UK); or GST, mAb 17A10 (1/100). This was followed by washing in PBS and incubation with secondary antibody (peroxidase-labelled rabbit anti-mouse immunoglobulins, P0260; 1/1000; Dako, Denmark). Alternatively, for the detection of annexin A5, pAb ANXA5 (Abcam; ab14196; 1.4 μg/mL) primary antibody followed by goat anti-rabbit Ig HRP (P0448; Dako; 1/1000) secondary antibody. All antibodies were diluted in PBS containing 0.05% Triton X, 0.1% BSA, 1% horse serum and 1% fetal bovine serum. XIRP1 and annexin A5 antibody reacting bands were detected with SuperSignal West Femto chemiluminescent reagent (Cat No: 34094; ThermoFisher Scientific) and GST antibody reacting bands were detected with SuperSignal West Pico Plus chemiluminescent reagent (Cat No: 34580; ThermoFisher Scientific) and visualized with a ChemiDoc Touch imaging system (BioRad Ltd.).

### Immunoprecipitation

Superparamagnetic polystyrene beads coated with monoclonal human anti-mouse IgG antibodies (Dynabeads Pan Mouse IgG; Cat No: 11041; Invitrogen) were washed with 4% bovine serum albumin in PBS and then incubated with mAb against POPDC1 (1C12) or PBS alone (control) with gentle rolling for 1 h. Beads were then washed with PBS and incubated with cultured human myotube extract in RIPA buffer for 1 h with rolling. Beads were thoroughly washed with RIPA buffer and then with PBS and the bound material extracted by incubating the beads with SDS sample buffer, heating to 90 °C for 3 min and then saving the eluate.

### Immunofluorescence microscopy

Immunohistochemistry was performed on unfixed cryostat sections of human skeletal muscle or rat cardiac ventricle muscle. Monoclonal antibodies against: POPDC1 (1C12; 1:2 dilution); POPDC2 (12G12 1:2 dilution); XIRP1 (Xin-alpha (D-8); sc-166,658; 1/100); Actinin (MANDYS 141, 4A12, 1/50 [50]); were diluted and incubated on specimens for 1 h. Specimens were then washed and incubated with rabbit polyclonal antibodies: Connexin 43 (GJA1, ab11370; Abcam; 1/1000); XIRP1 (Cat No: HPA016750; Sigma; 1/150); Annexin A5 (ANXA5, ab14196; Abcam; 1/50); POPDC1/BVES (Cat No: STJ22848; St. John’s Laboratory Ltd., London, E16 2RD; 1/100); Amphiphysin (ab244375; Abcam; 1/50). Following antibody incubation, specimens were washed with PBS and then incubated with 5 μg/ml of each secondary antibody which was either: goat anti-mouse ALEXA 488 (Cat No: A11029, Molecular Probes, Eugene, Oregon, USA) and goat anti-rabbit ALEXA 546 (Cat No: A11010), or with goat anti-mouse ALEXA 546 (Cat No: A11030) and goat anti-rabbit ALEXA 488 (Cat No: A11034). Secondary antibodies were diluted in PBS containing 1% horse serum, 1% fetal bovine serum and 0.1% BSA, and incubated on the sections for 1 h. Alternatively, for some immunofluorescent labelling of rat cardiac sections, a single primary (polyclonal) antibody then single goat anti-rabbit ALEXA 546 antibody were used, followed by ALEXA-488 Phalloidin (Cat No: A12383; Molecular Probes) to label filamentous actin. Finally, DAPI (at 200 ng/ml) was added for the final 10 min of incubation to counterstain nuclei before washing and mounting the sections (Hydromount; Merck). For protein competition experiments, monoclonal antibodies were pre-incubated with 1 mg/mL recombinant protein preparation for 1 h at room temperature. Images were acquired by sequential scanning with a Leica TCS SP5 spectral confocal microscope (63x/1.4 oil) (Leica Microsystems, Milton Keynes, UK), except the human heart images which were acquired with a Zeiss LSM780 confocal microscope (40x/1.3 oil) (Carl Zeiss Microscopy, Cambridge, UK.

## Supplementary Information

**Additional file 1.** Table; Unfiltered mass spectrometry results.

**Additional file 2.** Original uncropped blots used for Fig. 1.

## Data Availability

Mass spectrometry data generated and analysed during this study are available in the supplementary information file, “Additional file 1”. Monoclonal antibodies are available from the MDA monoclonal antibody resource, Oswestry, UK: http://www.glennmorris.org.uk/mabs/WCIND.htm POPDC nucleotide sequence data is available from GenBank with the following accession numbers: Mouse Bves/Popdc1 (NM_024285.3); Mouse Popdc2 (NM_001081984.2); Human BVES/POPDC1 (NM_007073.4); Human POPDC2 (NM_022135.4).

## Abbreviations

ACTC1: Actin Alpha Cardiac Muscle 1
ANXA5: Annexin A5
AV: Atrioventricular
BIN1: Bridging Integrator 1 (also known as Amphiphysin II)
BSA: Bovine serum albumin
BVES: Blood Vessel Epicardial Substance (also known as POPDC1)
cAMP: Cyclic adenosine 3′,5′-monophosphate
CAV3: Caveolin 3
Cdk4: Cyclin-dependent kinase-4
CMYA1: Cardiomyopathy-associated protein 1 (also known as XIRP1)
c-Myc: MYC Proto-Oncogene
COS-7: African green monkey kidney fibroblast-like cell line
Cx43: Connexin-43 (Gap Junction Protein Alpha 1, GJA1)
DAPI: 4′,6-diamidino-2-phenylindole
FLAG: DYKDDDDK-tag
FRET: Fluorescence resonance energy transfer
GEFT: ARHGEF25 or Rho Guanine Nucleotide Exchange Factor 25
GST: Glutathione S-transferase
HEK293: Human embryonic kidney 293 cells
HRP: Horseradish peroxidase
hTERT: Human telomerase reverse transcriptase
IPTG: Isopropyl beta-D-1-thiogalactopyranoside
KCNK2: Potassium Two Pore Domain Channel Subfamily K Member 2 (TREK-1)
LGMD: Limb-girdle muscular dystrophy
mAb: Monoclonal antibody
Nano LC MSMS: Nanoscale liquid chromatography tandem mass spectrometry
NDRG4: NDRG Family Member 4 (or N-Myc Downstream-Regulated Gene 4 Protein)
pAb: Polyclonal antibody
PBS: Phosphate-buffered saline
PCR: Polymerase chain reaction
PMSF: Phenylmethylsulfonyl fluoride (serine protease inhibitor)
POPDC1: Popeye Domain Containing 1 (also known as BVES)
POPDC2: Popeye Domain Containing 2
POPDC3: Popeye Domain Containing 3
PR61-alpha: Protein Phosphatase 2 Regulatory Subunit B’Alpha (PPP2R5A)
RIPA buffer: Radioimmunoprecipitation assay buffer
SDS: Sodium dodecyl sulfate
TNE buffer: Tris-HCl, NaCl, EDTA buffer
TREK-1: Potassium Two Pore Domain Channel Subfamily K Member 2 (KCNK2)
VAMP2: Vesicle Associated Membrane Protein 2
VAMP3: Vesicle Associated Membrane Protein 3
XIRP1: Xin Actin Binding Repeat Containing 1 (also known as XIN or CMYA1 - cardiomyopathy-associated protein 1)
YFP: Yellow fluorescent protein
ZO-1: Zonula Occludens Protein 1 (Tight Junction Protein 1)
