# Supplementary material for: An interaction of heart disease-associated proteins POPDC1/2 with XIRP1 in transverse tubules and intercalated discs
Source: BMC Mol Cell Biol. 2020 Dec 1;21:88. doi: 10.1186/s12860-020-00329-3 (PMC7709239; doi:10.1186/s12860-020-00329-3)
Supplement: Supplementary file 2 — Additional file 2. Original uncropped blots used for Fig. 1. [file 12860_2020_329_MOESM2_ESM.pdf]

An interaction of heart disease-associated proteins POPDC1/2 with XIRP1 in transverse tubules and intercalated discs

Ian Holt, Heidi R Fuller, Roland FR Schindler, Sally L Shirran, Thomas Brand and Glenn E Morris

Additional File 2

Original uncropped blots used for Figure 1:

Vertical black arrows show the lanes of these uncropped blots that were used to make up Figure 1. Non-adjacent lanes in the original blot that were juxtaposed in Figure 1 are shown separated by a black line in figure 1.

Figure 1A original upper blot:

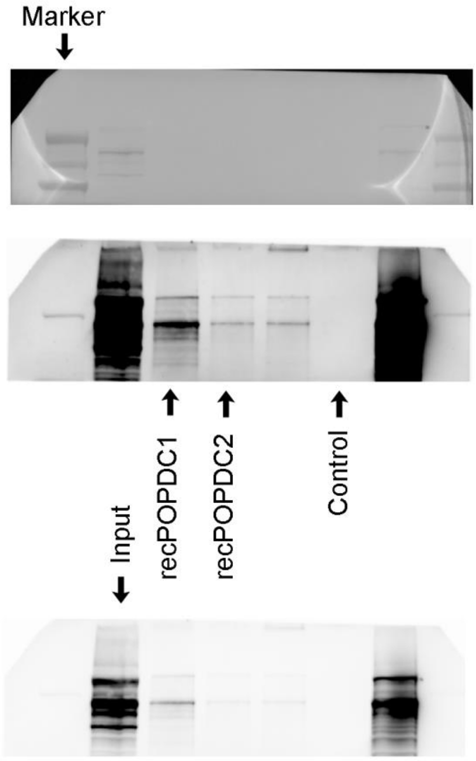

Colourimetric image:
Molecular weight marker

Chemiluminescent images (XIRP1):
Long exposure to show “recPOPDC1”, “recPOPDC2” and “Control” lanes

Short exposure to show “Input” lane

Figure 1A original lower blot:

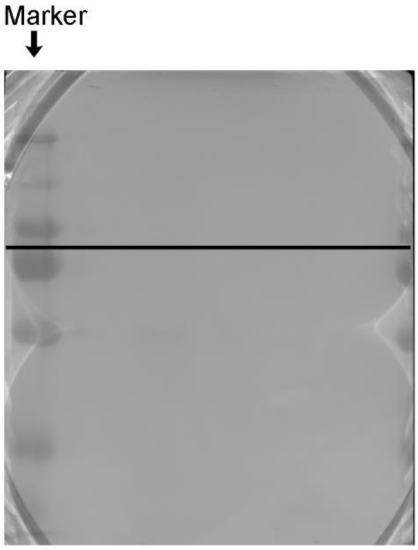

Image shown in Fig. 1A below the horizontal black line

Colourimetric image:
Molecular weight marker

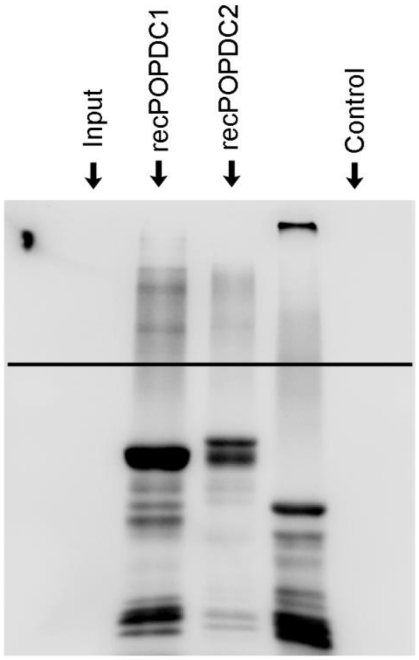

Chemiluminescent image (GST):
“Input”, “recPOPDC1”, “recPOPDC2” and “Control” lanes

Figure 1B original blot:

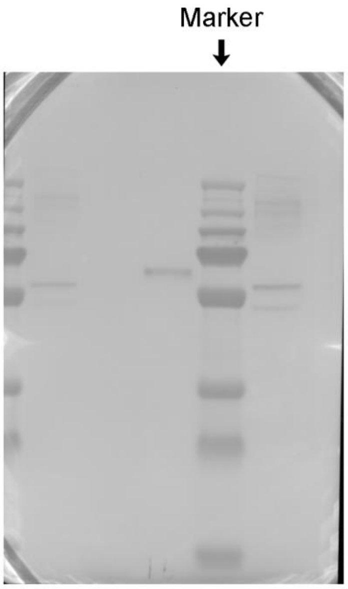

Colourimetric image:
Molecular weight marker

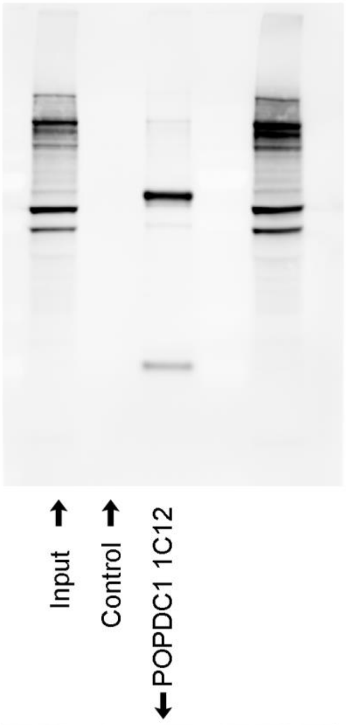

Chemiluminescent images (XIRP1):
Short exposure to show “Input” and “Control” lanes

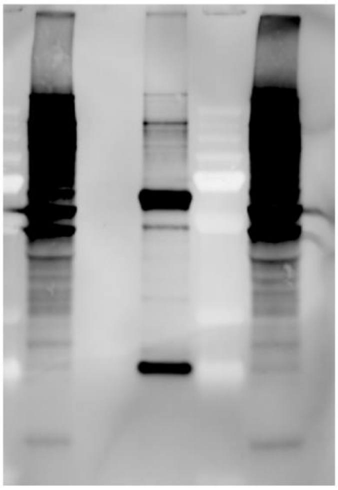

Long exposure to show “POPDC1 1C12” lane

Figure 1C original blot:

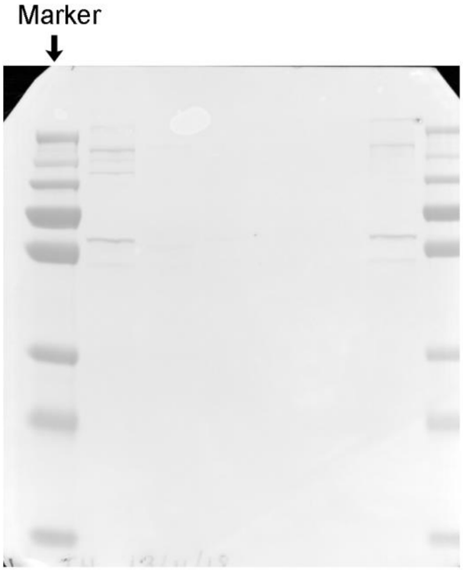

Colourimetric image:
Molecular weight marker

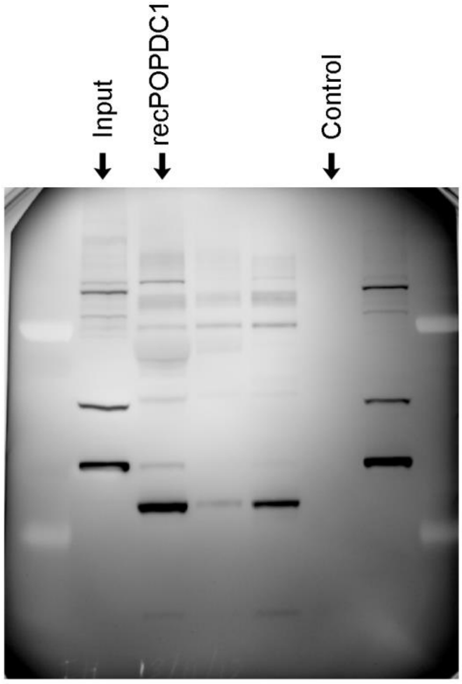

Chemiluminescent image (Annexin A5):
“Input”, “recPOPDC1” and “Control” lanes

Figure 1D original blot:

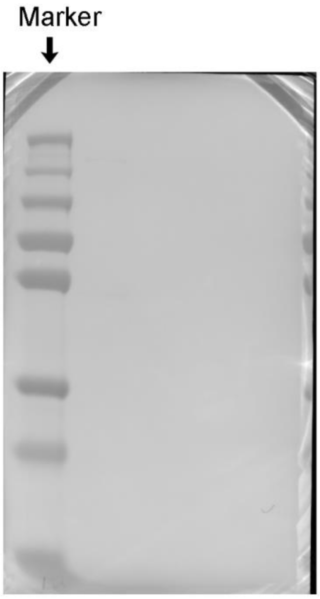

Colourimetric image:
Molecular weight marker

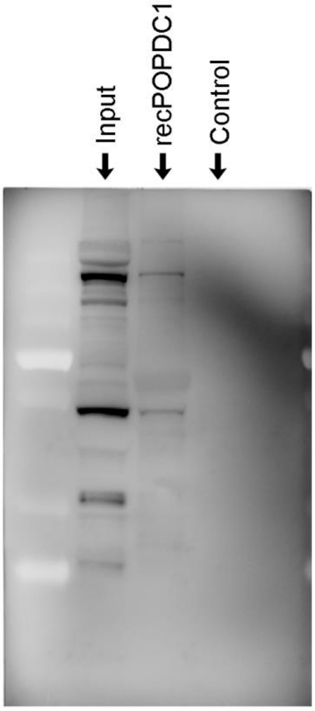

Chemiluminescent images (XIRP1):
“Input”, “recPOPDC1” and “Control” lanes
